# The Messages Presented in Electronic Cigarette–Related Social Media Promotions and Discussion: Scoping Review

**DOI:** 10.2196/11953

**Published:** 2019-02-05

**Authors:** Kahlia McCausland, Bruce Maycock, Tama Leaver, Jonine Jancey

**Affiliations:** 1 Collaboration for Evidence, Research and Impact in Public Health School of Public Health Curtin University Bentley Australia; 2 School of Media, Creative Arts and Social Inquiry Curtin University Bentley Australia

**Keywords:** electronic nicotine delivery systems, social media, public health, review

## Abstract

**Background:**

There has been a rapid rise in the popularity of electronic cigarettes (e-cigarettes) over the last decade, with growth predicted to continue. The uptake of these devices has escalated despite inconclusive evidence of their efficacy as a smoking cessation device and unknown long-term health consequences. As smoking rates continue to drop or plateau in many well-developed countries, transnational tobacco companies have transitioned into the vaping industry and are now using social media to promote their products. Evidence indicates e-cigarettes are being marketed on social media as a harm reduction alternative, with retailers and manufacturers utilizing marketing techniques historically used by the tobacco industry.

**Objective:**

This study aimed to identify and describe the messages presented in e-cigarette–related social media (Twitter, YouTube, Instagram, and Pinterest) promotions and discussions and identify future directions for research, surveillance, and regulation.

**Methods:**

Data sources included MEDLINE, Scopus, ProQuest, Informit, the *Journal of Medical Internet Research*, and Google Scholar. Included studies were published in English between 2007 and 2017, analyzed content captured from e-cigarette–related social media promotions or discussions, and reported results for e-cigarettes separately from other forms of tobacco and nicotine delivery. Database search ceased in October 2017. Initial searches identified 536 studies. Two reviewers screened studies by title and abstract. One reviewer examined 71 full-text articles to determine eligibility and identified 25 studies for inclusion. This process was undertaken with the assistance of the Web-based screening and data extraction tool—Covidence. The review was registered with the Joanna Briggs Institute (JBI) Systematic Reviews database and followed the methodology for JBI Scoping Reviews.

**Results:**

Several key messages are being used to promote e-cigarettes including as a safer alternative to cigarettes, efficacy as a smoking cessation aid, and for use where smoking is prohibited. Other major marketing efforts aimed at capturing a larger market involve promotion of innovative flavoring and highlighting the public performance of vaping. Discussion and promotion of these devices appear to be predominantly occurring among the general public and those with vested interests such as retailers and manufacturers. There is a noticeable silence from the public health and government sector in these discussions on social media.

**Conclusions:**

The social media landscape is dominated by pro-vaping messages disseminated by the vaping industry and vaping proponents. The uncertainty surrounding e-cigarette regulation expressed within the public health field appears not to be reflected in ongoing social media dialogues and highlights the need for public health professionals to interact with the public to actively influence social media conversations and create a more balanced discussion. With the vaping industry changing so rapidly, real-time monitoring and surveillance of how these devices are discussed, promoted, and used on social media is necessary in conjunction with evidence published in academic journals.

## Introduction

There has been a dramatic rise in the popularity of electronic cigarettes (e-cigarettes) since the first commercialized product was developed in China in 2003 [1,2]. It is now estimated that there are 35 million e-cigarette users globally (including heat not burn tobacco products) [3], with this rapid growth predicted to continue. According to BIS Research [4], the global e-cigarette industry will experience an annual growth of more than 22% until 2025, reaching a total market value of US $50 billion dollars at this time.

Since the advent of first generation e-cigarettes, which closely resemble traditional cigarettes in appearance and size, they have been the center of much debate. It has been suggested that these devices may be a less harmful alternative to smoking [5], provide health benefits to smokers who switch completely to them [6], lessen cigarette cravings [7], and facilitate smoking cessation [8]. However, promotion of e-cigarettes may also encourage nonsmokers, particularly young people, to initiate use [9,10], facilitate experimentation with traditional tobacco products [11], and undermine tobacco control efforts [12]. Recent studies also suggest that e-cigarette use is associated with negative health consequences [13,14] and may not facilitate adult smokers to quit at rates higher than smokers who do not use these products [15]. These contrasting arguments are evident in Web-based marketing by e-cigarette retailers and manufacturers [16], along with social media discussions about e-cigarettes [17]. Furthermore, the lack of agreement among countries on the population-level impact of these devices and how they should be regulated [2] (eg, UK Royal College of Physicians identifies e-cigarettes as a public health strategy, whereas the World Health Organization and the US Surgeon General see them as presenting potentially new health problems [18]) may cause confusion among consumers and the public in general. This, therefore, highlights the importance of examining social media as it offers opportunities to attract new users, promote continued use, and build brand loyalty.

Traditionally dominated by small start-up companies, the e-cigarette market has experienced rapid growth and transition, and more recently, large manufacturers and transnational tobacco companies have come to dominate the market. Major tobacco companies have entered the vaping industry by either acquiring e-cigarette companies and brands or developing their own products. Major tobacco companies now involved in the vaping industry include British American Tobacco, Imperial Tobacco, the Altria Group, Reynolds American, Philip Morris International, and Japan Tobacco International [19]. These companies have benefited from large advertising and marketing budgets, which enable promotion across the World Wide Web [20].

A significant portion of e-cigarette business is conducted on the internet [21], with most existing e-cigarette companies operating websites or other Web-based selling systems [22]. Sources suggest that e-cigarette manufacturers are careful to distance their products from tobacco [23] by using techniques such as aesthetic appeal, including attractiveness, coolness, colors, and innovative packaging and flavor variations. In addition, websites and social media accounts have been found to exhibit price promotions, and competitions and discount coupons; there is also evidence of celebrity endorsements and sports sponsorship [24].

An accurate understanding of the types of e-cigarette messages social media users are exposed to, and who is disseminating this information can assist in the development of appropriate surveillance to inform future policy and regulations. A scoping review was, therefore, undertaken to identify and describe the messages presented in e-cigarette–related social media (Twitter, YouTube, Instagram, and Pinterest) promotions and discussions.

## Methods

### Scoping Review Overview

The review was registered prospectively with the Joanna Briggs Institute (JBI) Systematic Reviews database (May 5, 2017) and proposed methods specified in advance in a protocol [25]. The scoping review adhered to the methods manual developed by the JBI [26].

### Objectives

This scoping review aimed to identify and describe the messages presented in e-cigarette–related social media (Twitter, YouTube, Instagram, and Pinterest) promotions and discussions and identify future directions for research, surveillance, and regulation.

### Inclusion Criteria

Included studies had to examine and analyze e-cigarette–related social media promotions and discussions. Studies needed to clearly identify the social media platform under investigation. Studies reporting multiple social media platforms were excluded unless results for each platform were reported separately. This was so the results for each social media platform could be extracted and reported, making it possible to clearly identify similarities and differences between the platforms. Studies identifying other tobacco products (eg, tobacco cigarette, snus, chewing tobacco, or hookah) were excluded unless e-cigarettes were also examined and reported separately. In addition, studies that did not distinguish between e-cigarettes and other forms of tobacco and nicotine delivery were excluded. Studies examining traditional media (eg, television and newspaper) were excluded unless social media platforms were also examined and reported separately. Studies were limited to the following countries: the United Kingdom, the Unites States of America, New Zealand, Australia, and Canada. These countries were selected as they are all developed countries and e-cigarette use is well established [27]. The review considered only peer-reviewed primary research studies published in English in the last 10 years (2007-2017); this period correlates with the approximate time that e-cigarettes were first introduced to the Unites States and Europe [28].

**Table 1:** Summary of excluded studies subject to full-text review with reason (N=48).

Reason for exclusion		Studies (n)
**Excluded at full-text review**		
	Wrong study design (ie, does not examine a social media platform or code for account type, theme, or sentiment)	12
	Does not report electronic cigarettes (e-cigarettes) in the results	7
	Results for different social media platforms not reported separately	2
	Publication type	4
	Country of study	1
**Excluded at data extraction**		
	Wrong study design	14
	Results for e-cigarettes not reported separately	2
	Results for different social media platforms not reported separately	1
	A specific population is examined (ie, people with mental illness)	2
	Country of study	3

### Search Strategy and Study Selection

Overall, 5 databases were searched (MEDLINE, Scopus, ProQuest, Informit, and Google Scholar) using the following terms:

(“electronic cigarette” OR e-cigarette OR “electronic nicotine delivery system” OR “personal vapo?ri?er” OR “electronic nicotine delivery device” OR “vape pen” OR “smokeless tobacco” OR “electric cigarette” OR “electric nicotine delivery system” OR “electric nicotine delivery device” OR e-hookah OR e-juice OR e-liquid OR vaping) AND (“social media” OR internet OR online OR YouTube OR Facebook OR Instagram OR Twitter OR “online media” OR “digital media” OR “social networking”) AND (“content analysis” OR “content evaluation” OR message OR meaning OR coding OR “media analysis” OR “textual analysis”).

In addition, the search strategy was entered as a nested Boolean search into Google Scholar, with the first 200 results examined for eligibility and subject to the screening process outlined below. Preliminary searches located relevant studies published in the *Journal of Medical Internet Research*, a hand-search of this journal was, therefore, also undertaken.

Retrieved references from each database were imported into EndNote X7 (Clarivate Analytics) [29] reference management software, with duplicate references removed before being imported into Covidence [30]. Covidence is a Web-based software platform that streamlines the production of systematic reviews by supporting the key steps in the review process [30]. Studies were assessed for inclusion, examined initially by title and abstract. Full-text articles were retrieved for those studies that appeared to meet the inclusion criteria or if further examination was required to determine eligibility. Moreover, 2 reviewers (KM and JJ) independently screened all titles and abstracts to determine their eligibility. The primary reviewer (KM) then undertook full-text screening. These processes were assisted by the Web-based screening and data extraction tool—Covidence [30]. Finally, the reference list of all articles subject to full-text review was screened to determine possible inclusion of additional studies. Identified studies were assessed for suitability based on full-text review undertaken by the primary reviewer. A summary of excluded studies subject to full-text review and the reason for exclusion is provided in Table 1.

### Extraction of Results

The relevant content from each study was extracted using a data extraction pro forma, which included title, author, publication year, country of study, aim/purpose of study, social media platform, sample size, study design/methods, results, and key findings that relate to the review question. Included studies were required to have developed coding categories for content including one or more of the following: account type, themes, and sentiment. Account type characterizes the publisher of the social media post; theme reflects the domain of the actual content conveyed, such as the categories of health, smoking cessation, and regulation; and sentiment reflects the stance expressed in a social media post toward e-cigarettes, related products or its users, whether positive, neutral, or negative. To ensure data extraction consistency, 2 reviewers (KM and JJ), independent of one another, extracted data from the same 5 studies using the data extraction pro forma. The reviewers then met to determine whether the extraction approach was consistent. The primary reviewer (KM) then went on to extract data from the remaining studies unaccompanied.

## Results

### Description of Included and Excluded Studies

**Figure 1:**
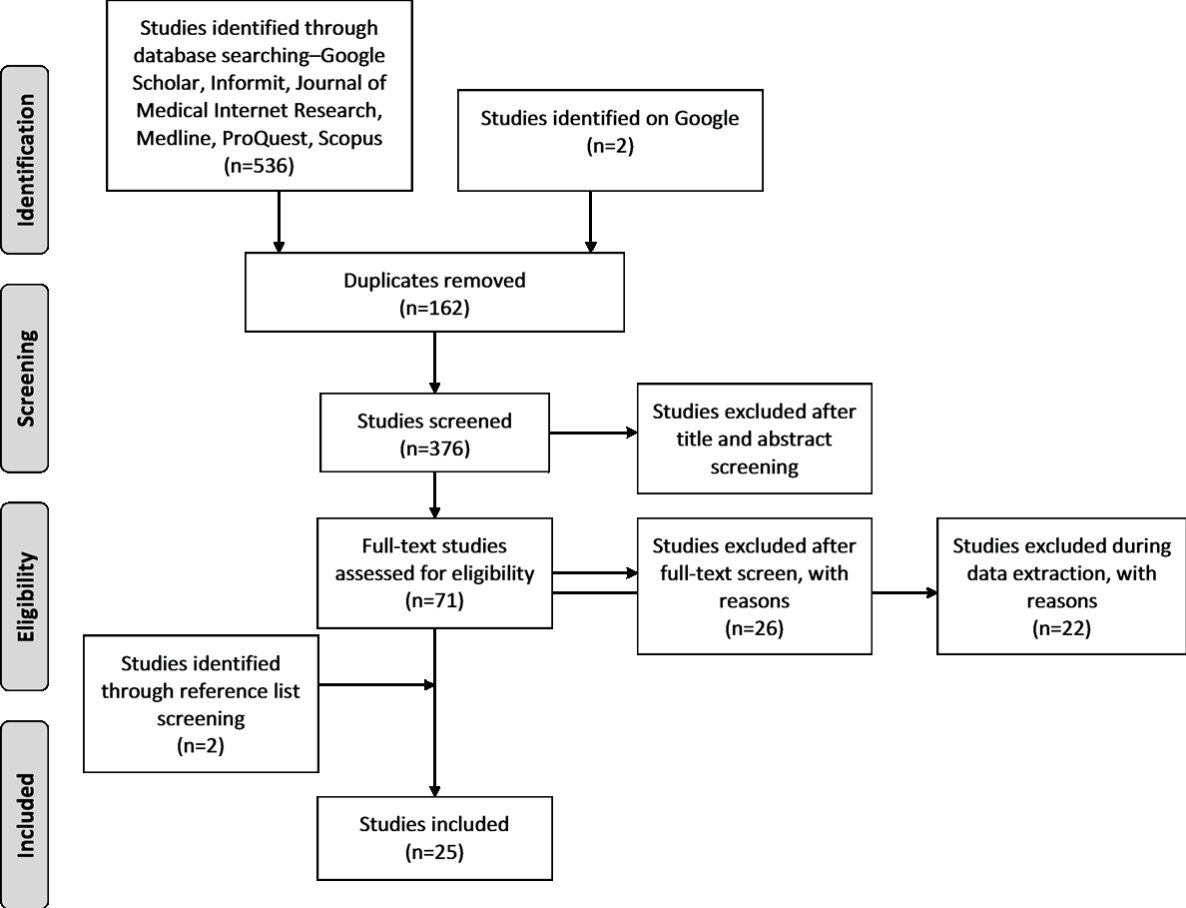
Preferred Reporting Items for Systematic Reviews and Meta-Analyses flow diagram.

The Preferred Reporting Items for Systematic Reviews and Meta-Analyses flow diagram detailed in Figure 1 presents the number of studies at each stage of the review process.

A total of 25 studies were identified for inclusion in this review. A total of 18 studies analyzed Twitter data [16,17,31-46]; 4 examined YouTube including videos [47-49] and data associated with videos, such as video tags, titles, or descriptions [50]; and 3 studies investigated images on Instagram and Pinterest [51-53].

Twenty-four studies were conducted in the United States [16,17,31-41,43-53] and one in Canada [42] (Table 2).

### Sample Size and Data Coding

The sample size of included studies varied widely, even within social media platforms (Twitter, YouTube, Pinterest, and Instagram), with the platform under investigation influencing the coding method used (Table 2). Methods used included hand coding [31,32,36-39,41,42,46-53] or machine learning [44], or a combination of the 2 methods [16,17,33-35,40,43,45]. Hand coding involved one or more human coders categorizing data, whereas machine learning used algorithms to give computers the ability to code data, although hand coding was usually used for an initial subset of data to help refine the algorithm to improve its accuracy [16,33-35,43,45]. Studies that analyzed text (ie, tweets from Twitter and YouTube video descriptions) predominantly employed hand coding for smaller samples (eg, <3000) [31,32,36-39,42,46], and a combination of hand coding and machine learning [16,33-35,40,43,45] or only machine learning [44] for larger samples, the largest being 1,669,123 tweets. Three studies did, however, hand code samples of over 10,000 [17,41,50]. All studies that analyzed images (ie, Pinterest, Instagram, and YouTube) did so by hand coding samples of between 46 and 2208 posts and videos (Table 2).

### Account Type

A total of 12 studies [16,31,33,37,39,41-44,47-48,53] used coding to identify the source (account type) of the social media data collected in their sample, most commonly informed by data found in account profiles (eg, bio, location, and profile photo) and preceding social media posts (Table 3). The most common account types coded for were personal [16,31,33,39,41,42,44,47,48,53] and commercial [33,37,39,41, 42,44,48,53], of which these account types represented up to 82.68% (104,283/126,127) [44] and 89.73% (66,102/73,672) [33] of some samples, respectively. Overall, 3 studies coded for government, foundation or not for profit organizations, [39,41] and public health and health care [42] accounts. All were studies of Twitter discussions that unanimously reported that tweets from these account types represented less than 3% of their sample size (1.0%, 5/500 for [39]; 0.08%, 8/10,128 for [41], and 1.3%, 4/300 and 3.3%, 10/300 for [42]). Overall, 3 studies coded for fake accounts, of which 2 reported these accounts represented similar percentages of their sample (6.90%, 699/10,128 for [41] and 9.7%, 29/300 for [42]), whereas the third found an overabundance (80.7%, n not provided) [16].

**Table 2:** Description of included studies, sample size, and coding method.

Authors, year, country	Social media platform	Sample size	Coding method
Burke-Garcia et al, 2017, United States [39]	Twitter	1000 tweets	Hand coding
Lazard et al, 2017, United States [40]	Twitter	4629 tweets	Machine learning and hand coding
Allem et al, 2017, United States [31]	Twitter	2192 tweets	Hand coding
Ayers et al, 2017, United States [17]	Twitter	11,600 tweets	Hand coding
Dai et al, 2017, United States [45]	Twitter	757,167 tweets	Hand coding and machine learning
Clark et al, 2016, United States [16]	Twitter	850,000 tweets	Hand coding, machine learning, and hedonometrics
van der Tempel et al, 2016, Canada [42]	Twitter	600 tweets	Hand coding
Han et al, 2016, United States [35]	Twitter	1,021,561 tweets	Hand coding and machine learning
Jo et al, 2016, United States [36]	Twitter	2847 tweets	Hand coding
Kavuluru et al, 2016, United States [43]	Twitter	224,000 tweets	Hand coding and machine learning
Sowles et al, 2016, United States [37]	Twitter	1156 tweets	Hand coding
Unger et al, 2016, United States [38]	Twitter	1519 tweets	Hand coding
Lazard et al, 2016, United States [44]	Twitter	126,127 tweets	Machine learning
Cole-Lewis et al, 2015, United States [41]	Twitter	10,128 tweets	Hand coding
Kim et al, 2015, United States [34]	Twitter	1,669,123 tweets	Hand coding and machine learning
Harris et al, 2014, United States [32]	Twitter	683 tweets	Hand coding
Huang et al, 2014, United States [33]	Twitter	73,672 tweets	Handing coding and machine learning
Prochaska et al, 2012, United States [46]	Twitter	153 accounts	Hand coding
Sears et al, 2017, United States [47]	YouTube	46 videos	Hand coding
Basch et al, 2016, United States [48]	YouTube	99 videos	Hand coding
Merianos et al, 2016, United States [49]	YouTube	55 videos	Hand coding
Huang et al, 2016, United States [50]	YouTube	28,089 videos tags, titles, or descriptions	Hand coding
Lee et al, 2017, United States [51]	Instagram and Pinterest	1800 images	Hand coding
Chu et al, 2016, United States [52]	Instagram	2208 posts	Hand coding
Laestadius et al, 2016, United States [53]	Instagram	85 posts	Hand coding

### Themes

All 25 included studies coded for themes (Table 4). Health, safety, and harms was the most coded for theme in this review [17,31-33,35,38,39,41,43,46-51,53]; however, various descriptions for health, safety, and harms were used (eg, health, harm reduction, and harms encompassing both the health benefits and consequences of e-cigarette use). Additional themes frequently cited were smoking cessation [16,17,33,35,37, 39,41-43,46,47,49,50,53]; product types and characteristics [16,17,32-37,39,41,43,49-51]; advertisement, promotion, and marketing [16,31,38,39,41,42,44,45,48,51,52]; regulation, policy, and government [31,32,35,39-42,49,50]; price promotions, discounts, coupons, giveaways, and competitions [16,33,34,36,37,44,50]; and smoke-free, use indoors or where cigarettes are banned [17,35,40,43,47,49].

### Sentiment

Of the 25 studies, a total of 12 coded for sentiment [16,31,32,38-43,45,47,49] (Table 5). Overall, 3 studies made the distinction when coding for message attitude [38,42,45] rather than emotional tone or affective content.

**Table 3:** Coded category—account type.

Account type	Studies, n (%)	References
Personal (general public, individuals, organic, and user-generated)	10 (40)	[16,31,33,39,41,42,44,47,48,53]
Commercial (marketing, tobacco or electronic cigarette [e-cigarette] company or retailer)	8 (32)	[33,37,39,41,42,44,48,53]
Press, media, or news (verifiable press or other prominent media sources of information, such as blogs)	3 (12)	[41,42,48]
Fake (hacked, bots, and automated)	3 (12)	[16,41,42]
Professional (television studio or network, production company, or organization)	2 (8)	[47,48]
Government, foundation, or not for profit organization	2 (8)	[39,41]
Proponents (sales or marketing agencies and individuals who advocate or specifically identify themselves as vapers)	2 (8)	[43,44]
Celebrity or public figure	2 (8)	[41,42]
Unknown or other	2 (8)	[31,37]
Public health, health care	1 (4)	[42]
Vaping-related handle (vaping-related term in handle name or Twitter bio)	1 (4)	[37]
Personal accounts with industry ties	1 (4)	[42]
E-cigarette community movement	1 (4)	[41]
General entity (company, store, or advocacy group)	1 (4)	[31]

**Table 4:** Coded category—themes.

Themes		Studies, n (%)	References
**Health, safety, and harms**		16 (64)	[17,31-33,35,38,39,41,43,46-51,53]
	Health	10 (40)	[31,33,38,46-51,53]
	Safety	5 (20)	[17,32,33,48,50]
	Harms	2 (8)	[48,49]
	Harm reduction	2 (8)	[35,43]
	Health and safety	1 (4)	[41]
	Health and health consequence	1 (4)	[39]
Smoking cessation		14 (56)	[16,17,33,35,37,39,41-43,46,47,49,50,53]
Product types and characteristics		14 (56)	[16,17,32-37,39,41,43,49-51]
Advertisement, promotion, marketing		11 (44)	[16,31,38,39,41,42,44,45,48,51,52]
Regulation, policy, government		9 (36)	[31,32,35,39-42,49,50]
Price promotions, discounts, coupons, giveaways, competitions		7 (28)	[16,33,34,36,37,44,50]
Smoke-free, use indoors or where cigarettes are banned		6 (24)	[17,35,40,43,47,49]
More economical than smoking		5 (20)	[17,42,47,49,53]
Social status, acceptance		4 (16)	[17,38,47,51]
Cleaner than tobacco, environment friendly, no/minimal odor		4 (16)	[17,47,49,53]
First or second person experience, use, opinion, or purchases		4 (16)	[39,42,52,53]
Recreation, customization, tricks		3 (12)	[47,51,53]
Other/unknown		3 (12)	[31,38,39]
Product image		2 (8)	[37,52]
Craving		2 (8)	[41,42]
Illicit substance use in e-cigarettes		2 (8)	[41,51]
Personal opinion		2 (8)	[42,45]
News articles and updates		2 (8)	[42,44]
Tastes good		2 (8)	[42,49]
Getting others started, encouraging use, offering advice		2 (8)	[40,42]
Second-hand smoke		2 (8)	[47,49]
Cessation devices or gateway products for youth to establish nicotine addictions		2 (8)	[44,49]
Text		1 (4)	[52]
Lies/propaganda		1 (4)	[32]
Science (studies)		1 (4)	[32]
Issue salience		1 (4)	[32]
Underage e-cigarette use		1 (4)	[41]
E-cigarette use in association with other addictive substances (eg, alcohol, caffeine)		1 (4)	[41]
Parental e-cigarette use		1 (4)	[41]
Places of use		1 (4)	[34]
Neutral information		1 (4)	[42]
Humor		1 (4)	[42]
Just starting e-cigarettes		1 (4)	[42]
Advocating e-cigarettes		1 (4)	[42]
Attempt to engage other Twitter users		1 (4)	[42]
Using or comparing with other substances/nicotine replacement therapies		1 (4)	[42]
Presence of identity or community		1 (4)	[53]
Technology (modern products, information about science behind the products)		1 (4)	[47]
Celebrity, model		1 (4)	[51]
Meme		1 (4)	[51]
Anti-smoking		1 (4)	[51]
Utilization patterns		1 (4)	[39]
Consumer endorsement		1 (4)	[39]
Money (taxes, small businesses)		1 (4)	[31]
Addiction to e-cigarettes		1 (4)	[49]
Reactions to e-cigarette policies and questions about e-cigarette health risk claims		1 (4)	[44]
Similar to real cigarettes		1 (4)	[49]

**Table 5:** Coded category—sentiment.

Sentiment		Studies, n (%)	References
**Emotional tone or affective content**			
	Positive or negative	5 (20)	[16,41-43,47]
	Positive or negative valence	2 (8)	[39,40]
	Pro or anti	2 (8)	[31,49]
	Pro- or anti-policy	1 (4)	[32]
	Neutral	7 (28)	[31,39,41,42,45,47,49]
	Unable to tell	1 (4)	[32]
**Message attitude**			
	Pro or con	1 (4)	[42]
	Pro or anti	1 (4)	[38]
	Supportive or against	1 (4)	[45]
	Neutral or do not know	3 (12)	[38,42,45]

## Discussion

### Principal Findings

#### Data Coding

The coding methods used were hand coding, machine learning, or a combination of the two. Compared with hand coding, machine learning can rapidly code large amounts of data; however, hand coding undertaken by humans may more accurately discriminate the complexities and subtleties of language [54]. Although hand coding can be subject to individual bias, the development of codes grounded in literature and achieving acceptable levels of inter-rater reliability can assist to reduce this [55]. Studies that require the determination of subtle differences in language or context may, therefore, be better placed to employ hand coding for a smaller sample of data, whereas studies that rely less on context could employ machine learning to code larger samples [55]. The increased complexity of interpreting visual social media (eg, YouTube, Instagram, and Pinterest) meant all studies of these platforms employed hand coding [47-49,51-53].

### Account Type

#### Personal

Studies included in this review reported dissemination of diverse e-cigarette messaging by predominantly commercial social media accounts [33,53]; however, other studies discovered conversations occurring among personal accounts dominating the social media landscape [31,41,42,44,47,48]. Personal accounts were found to be discussing, endorsing, and promoting various themes, most commonly marketing [41,48,53], smoking cessation [33,42,44], recreation and technology [47,53], and first-person experience and opinion [41,42]. This is particularly important as individuals may be less critical of material posted by other consumers compared with retailers [56,57] and may be more easily persuaded by other individuals they know, given their relative closeness and potentially increased perception of source credibility [58,59].

#### Commercial

Several key marketing strategies were found to be used by commercial social media accounts. These included the use of popular hashtags that enabled marketing messages to *piggy back* on trending topics and increase dissemination reach [42], use of fake user accounts to disseminate spam and favorable views [33,42], and the offer of price promotions and product giveaways [33,44,53]. Social media networking and marketing efforts undertaken by the vaping industry may have contributed to the rapid rise in popularity of e-cigarettes, the extent of which has been demonstrated by the findings in this review. It has also been hypothesized by some researchers that the lack of regulatory standards on social media may be playing an ever-increasing role in the diffusion of tobacco products and prosmoking messages [60].

#### Government, Foundation or Not for Profit Organizations, and Public Health and Health Care

Of the studies that coded for government, foundation, or not for profit accounts [39,41,42], limited public health–related messaging was identified, and activity from these account types represented less than 3% of samples. These findings indicate more action from public health and government to communicate the potential harms and benefits of alternate nicotine delivery products via social media is required to balance the information shared on these platforms.

#### Fake

Most tweets produced by accounts classified as fake were found to promote e-cigarettes as effective smoking cessation aids, either by emulating first-person anecdotes or linking to news articles or other Web-based media [41,42], with some accounts potentially using computer programs to generate and post content automatically [33,34].

The general tweet structure from an automated bot is provided here [16]:

@USER [I,We] [tried, pursued] to [give up, quit] smoking. Discovered BRAND electronic cigarettes and quit in [#] weeks. [Marvelous,Amazing,Terrific]! URL

@USER It’s now really easy to [quit,give up] smoking (cigarettes).—these BRAND electronic cigarettes are lots of [fun,pleasure]! URL

@USER electronic cigarettes can assist cigarette smokers to quit, it’s well worth the cost URL

This type of spamming suggests that there are remunerations to be gained by steering potential online consumers to certain retail websites [34].

### Themes

#### Health, Safety, and Harms

All studies that coded for health, safety, and harms reported that e-cigarettes are being referred to as *healthier* and *safer* than traditional tobacco products on social media [17,31-33,35,38,39,41,43,46-51,53]. Provided that scientific evidence about the safety of e-cigarettes is largely inconclusive, marketing claims that use words such as *safer* to describe their products could contribute to confusion about their overall safety, especially among youth. Promoting a product by claiming that it is healthier than tobacco smoking, the leading cause of preventable death, is therefore controversial and may only have merit when targeting smokers who are contemplating quitting or reducing use [61].

There is indication that an individual’s perception of a substance’s potential harms and benefits and their behavior of use is influenced by the availability of information discussing the health effects of that substance [62]. A recent analysis reports that 34.20% (8433/24,658) of American youth sampled believe that e-cigarettes are less harmful than cigarettes, and 45.00% (11,096/24,658) are not sure [63].

Example *safety* coded tweets are displayed in the following excerpt [32]:

RT @ChiPublicHealth: Electronic cigs contain a dangerous, addictive drug & should be regulated like other nicotine products #ecigtruths

@ChiPublicHealth it’s not about being safe, it’s about being SAFER than the alternative #EcigsSaveLives it’s about HARM REDUCTION #Casaa

#### Smoking Cessation

Over half (56%, 14/25) of studies included in this review found evidence of e-cigarettes being promoted as a smoking cessation tool [16,17,33,35,37,39,41-43,46,47,49,50,53], although their efficacy as such is yet to be determined [8]. However, some research indicates much smaller proportions of e-cigarette advertisements are now endorsing these devices as quit aids [37,42], and cited reasons for use by vapers have significantly shifted away from smoking cessation (43.00%, 1247/2900 in 2012 vs 29.00%, 841/2900 in 2015) toward use to increase social image (21.00%, 609/2900 in 2012 vs 37.00%, 1073/2900 in 2015) [17]. Of concern is that these results suggest that e-cigarette uptake is not solely driven by a desire among smokers to quit smoking [64].

#### Product Types or Characteristics

Overall, 14 studies coded for e-cigarette product characteristics such as brands, flavors, and nicotine content, and of these, the majority (86%, 12/14) [16,17,32,35-37,39,41,43,49-51] coded for the mention or depiction of electronic cigarette juice (e-juice) flavors. Marketing social media posts and videos were most commonly found to promote the vast array of e-juice flavors available on the market [16,35,37,43,49], a strategy historically used to entice new tobacco consumers [65], especially youth [66]. As a result of mounting evidence that flavored tobacco products facilitate youth smoking [67], these products (aside from menthol) were effectively banned in 2009 [68]. However, no such ban currently exists for e-cigarettes with thousands of flavors available for purchase [22]. Some research suggests that flavors appeal to adult smokers and may aid smoking cessation [69,70]; nevertheless, increasing evidence demonstrating that flavors also attract youth to the e-cigarette market is mounting [71-73], which is a cause for concern as nicotine addiction has been found to cause problems with adolescent brain development [74]. Studies have found flavor profiles (eg, tobacco and menthol) that are more appealing to some adults may have minimal appeal to young people [69,75]. It has, therefore, been proposed as a harm reduction strategy that these flavors be promoted to adults to assist tobacco substitution, whereas restricting those flavors that appeal most to youth (eg, fruits and deserts) [76].

#### Advertisement, Promotion, and Marketing

A concern of e-cigarette promotion on social media is the high level of cross-platform interaction (ie, using apps to post content across several social media platforms) [33], and given the sizeable youth presence on these platforms [77] provides an avenue to invite nonsmokers, youth in particular, to experiment with and instigate use. However, just because youth are utilizing social media does not inevitably mean they are subjected to e-cigarette marketing, as they would need to *follow* these accounts, be exposed through their social networks (ie, followers or those they are following), or browse via direct searches [34]. Recent studies have, however, found that e-cigarette users learn about vaping and these devices from the internet and social media [78,79]; therefore, monitoring how e-cigarettes are promoted on these platforms is incredibly important.

#### Regulation, Policy, and Government

Messages against government regulation were found to be most prominent [31], for example:

Wow, CA DPH thinks it acceptable to deceive the ppl it is supposed to serve: #stillblowingsmoke? no #notblowingsmoke Don’t let the FDA go without making your voice heard…#vapecommunity #vape #ecig #notblowingsmoke #ecigssavelive

Many antiregulation posts expressed concern over the motivations for wanting e-cigarettes regulated, suggesting policy makers were only concerned about these devices because tobacco revenue would decrease if people started using them and that policy represents the teaming of government and industry such that the Food and Drug Administration (FDA) deeming rule would work only to enhance the power of Big Tobacco [31,32,40,42,44]. The uncertainty surrounding e-cigarette regulation expressed within the public health field appears not to be reflected in ongoing social media dialogues [41] and highlights the need for public health professionals to interact with the public to actively influence social media conversations and create a more balanced discussion [40,44].

#### Price Promotions

This review provides evidence of the existence of e-cigarette marketing on social media, of which a substantial portion includes price promotions, discounts, coupons, free trials, giveaways, and competitions [16,33,34,36,37,44,50]. These types of incentives can persuade potential consumers to make a purchase and assist vendors to create a loyal customer base [80], which has already been demonstrated for tobacco [81,82]. It is well documented that smoking behaviors react to changes in cigarette prices [83], and in response, tobacco control efforts have sought to eradicate the use of these incentives [84]. Similarly, studies have reported that e-cigarette sales are very responsive to price variation, and implementing policy to limit price promotions, free-trials, and giveaways could lead to significant behavior change and uptake [85]. People who use e-cigarettes regularly cite smoking cessation as their motivation for vaping initiation; for this group of people, price promotions that enable affordability of these products longer term could be viewed as appropriate [37], although evidence supporting the use of these devices as a smoking cessation aid is still out for debate [8].

#### Smoke-Free and Use Indoors or Where Cigarettes Are Banned

A major drawback of cigarettes is the smoke they emit, which is known to contain thousands of chemicals dangerous to human health [86], and for this reason, cigarettes are now subject to smoking bans and smoke-free policies all over the world [87]. Studies included in this review found that e-cigarette proponents frequently highlight the smoke-free aspect of vaping and that these devices can be used where tobacco is currently restricted [17,35,40,43,47,49]. Marketing that accentuates that e-cigarettes can be used *anywhere* may undermine enforcement of smoke-free policies and tobacco control efforts [12] and expose nonusers to toxins [13]. A survey of a representative sample of American adults found that increased frequency of exposure to e-cigarette advertising was associated with lower support for policies that restrict use in public places [88]. These results suggest the need for more publicly available information regarding the chemical composition and possible health consequences of inhaling second-hand vapor [38].

#### Recreation

Less commonly coded for, however an important aspect of vaping to recognize is recreation, which was coded for among image-based social media (ie, Instagram, Pinterest, and YouTube) studies [47,51,53]. These studies commonly reported depictions of customization and modification of e-cigarette devices for both functional and aesthetic purposes and of vapers exhaling large plumes of vapor (known as cloud chasing) and performing vape tricks. Depiction of these vaping practices could contribute to the normalization of vaping, as images and videos represent these acts as fun and more commonplace and socially accepted than is in reality [52,89], with many posts accompanied by hashtags signifying community and social identity [53]. For example [31]:

What’s your favourite #vaping trick? #VapeTricks #Vapelife #VapeOn #NotBlowingSmoke

Many hashtags emerge from users themselves through an organic user-led process [90], with research suggesting substance –focused hashtags can serve as an “addiction bond” [91].

Social media posts and videos mentioning different product characteristics (eg, flavors, mods, and illicit substances) and displaying consumers’ ability to choose and modify aspects of their vaping experience indicates that customization and recreation largely contributes to e-cigarette discourse on social media and may have contributed to their rapid increase in popularity [47,50-52].

### Sentiment

Studies which coded for sentiment and did not specifically state they were coding for message attitude most commonly reported that their sample was positively skewed toward e-cigarettes, their users, and antiregulation [31,32,40-43,47,49], whereas studies that coded for message attitude reported predominantly neutral attitude [38,42,45]. Social media posts from accounts with vested interests (eg, commercial or automated) and the general public were found to present positive messages related to e-cigarettes [16,41-43,49], whereas news- and health-related accounts provided messages that were least positive or neutral [41,42,49].

Examples of positive, negative, and neutral tweets are provided here [39]:

Medical professionals surveyed. Overwhelmingly prefer #vaping to smoking. #vape #vaplyfe #the http://t.co/tcKsX6Dc0S http://t.co/tiJBNZjBZa

RT @StopVaping RETWEET this if your not VAPING today because you want to live.

Vaping in the United States has eclipsed cigarette smoking in some age groups. #Vaping #eCigarettes #Rosemont http://t.co/wzgVT0p2C1

The proliferation of social media platforms and *Big Data* analytics provides the opportunity to explore and monitor people’s perceptions of e-cigarettes in real time and what fuels opinion over time [41,45]. The studies included in this review could be used to establish a sentiment baseline for public health professionals to develop campaigns and interventions [41] and act as supplementary data to traditional surveys [45].

In agreement with Lienemann et al [55], when coding for sentiment, clarity and comparability across studies could be enhanced by distinguishing between attitude and emotion. For example, social media data can be provaping; however, it can have a negative emotional tone.

### Recommendations for Research

Given the volume of personal accounts found to be discussing, endorsing, and promoting various aspects of vaping, further research to determine who the *loudest* social media accounts are in the sense that their material is being seen and shared most frequently and how this material is influencing other social media users’ perceptions and use of e-cigarettes is, therefore, warranted [41]. The perceived safety of these products may also be a contributing factor in the increasing trend of vaping among adult never smokers and former smokers [92]. Research is, therefore, required to determine the implications of claims promoting e-cigarettes as a superior product on audience perception and use [47].

The use and depiction of vaping for recreation raises questions about the promotion of these devices as a hobby or socializing opportunity [64]. As such, it may be valuable to investigate the degree to which the vaping industry is targeting nonsmoking youth who may have an interest in vaping for enjoyment or as a hobby rather than a smoking cessation tool [37,93].

Furthermore, the US FDA has recognized the impact of e-cigarettes recently, ratifying a rule (August 8, 2016) that extended their regulatory authority to all tobacco products, including e-cigarettes. These regulations restrict youth access by prohibiting the sale of e-cigarettes to those aged under 18 years, embargos the use of free samples for promotion, and states e-cigarette products must now require a health warning [94]. These restrictions highlight the need for continued research and monitoring of social media commercialization of these products and for this issue to be placed on public health and policy agendas.

### Limitations

The review did not assess the quality of the evidence presented in each study, rather provided an overview regardless of quality as per the methodology outlined in the Manual for Scoping Reviews by JBI [26]. The search strategy included several popular terms used to describe e-cigarettes; however, keywords including emerging and variations of slang terms may have been overlooked and therefore, resulted in an incomplete retrieval of identified research. Furthermore, it is possible that additional studies relevant to the research question may have been identified if alternative databases were searched.

The reviewed material reflects a general bias toward certain social media platforms such as Twitter as its data are mostly public and easily accessible to researchers, whereas Facebook and other platforms are not [95]. This is not an indication that Facebook and other platforms are not spaces where e-cigarettes are discussed, but only that these activities are not visible to researchers.

### Conclusions

The social media landscape is being dominated by provaping messages disseminated by the vaping industry and vaping proponents, whereas the uncertainty surrounding e-cigarette regulation expressed within the public health field appears not to be reflected in ongoing social media dialogues. Latest generation e-cigarettes are resembling less and less their first generation *cig-a-like* counterparts and are being promoted not only as a smoking cessation device and safer alternative to smoking but also as a recreational activity whereby you can create your own unique vaping experience with the use of flavors, device modification, and vape tricks. With the industry changing so rapidly, real-time monitoring and surveillance of how these devices are discussed, promoted, and used on social media is necessary in conjunction with evidence published in academic journals. The need for real-time monitoring and surveillance also highlights the need to close the chasm between research and practice [96]. Some government agencies have recognized and are attempting to bridge this gap by introducing research translation initiatives, annual conferences, education programs, and more varied communications [97,98] as they attempt to move evidence through the publication pipeline faster and more efficiently. However, Departments of Health may well have to start thinking about investing in real-time monitoring and surveillance to interact with the public to actively influence social media conversations and create a more balanced discussion with regard to e-cigarettes.

## Abbreviations

e-cigarette: electronic cigarette
e-juice: electronic cigarette juice
FDA: Food and Drug Administration
JBI: Joanna Briggs Institute
